# Low-Temperature PECVD Growth of Germanium for Mode-Locking of Er-Doped Fiber Laser

**DOI:** 10.3390/nano12071197

**Published:** 2022-04-03

**Authors:** Chun-Yen Lin, Chih-Hsien Cheng, Yu-Chieh Chi, Sze Yun Set, Shinji Yamashita, Gong-Ru Lin

**Affiliations:** 1 Graduate Institute of Photonics and Optoelectronics, The Department of Electrical Engineering, National Taiwan University, Taipei 10617, Taiwan; r03941053@ntu.edu.tw (C.-Y.L.); d96941016@ntu.edu.tw (Y.-C.C.); 2 Research Center for Advanced Science and Technology, University of Tokyo, Tokyo 153-0041, Japan; f97941009@ntu.edu.tw (C.-H.C.); set@cntp.t.u-tokyo.ac.jp (S.Y.S.); syama@cntp.t.u-tokyo.ac.jp (S.Y.)

**Keywords:** ultra-thin Ge saturable absorber, mode-locked laser, Er-doped fiber laser

## Abstract

A low-temperature plasma-enhanced chemical vapor deposition grown germanium (Ge) thin-film is employed as a nonlinear saturable absorber (SA). This Ge SA can passively mode-lock the erbium-doped fiber laser (EDFL) for soliton generation at a central wavelength of 1600 nm. The lift-off and transfer of the Ge film synthesized upon the SiO_2_/Si substrate are performed by buffered oxide etching and direct imprinting. The Ge film with a thickness of 200 nm exhibits its Raman peak at 297 cm^−1^, which both the nanocrystalline and polycrystalline Ge phases contribute to. In addition, the Ge thin-film is somewhat oxidized but still provides two primary crystal phases at the (111) and (311) orientations with corresponding diffraction ring radii of 0.317 and 0.173 nm, respectively. The nanocrystalline structure at (111) orientation with a corresponding d-spacing of 0.319 nm is also observed. The linear and nonlinear transmittances of the Ge thin-film are measured to show its self-amplitude modulation coefficient of 0.016. This is better than nano-scale charcoal and carbon-black SA particles for initiating the mode-locking at the first stage. After the Ge-based saturable absorber into the L-band EDFL system without using any polarized components, the narrowest pulsewidth and broadest linewidth of the soliton pulse are determined as 654.4 fs and 4.2 nm, respectively, with a corresponding time–bandwidth product of 0.32 under high pumping conditions.

## 1. Introduction

Passively mode-locked fiber lasers self-started by exploiting a variety of saturable absorbers (SAs) have been investigated for decades [1,2,3,4,5,6,7,8,9,10]. These SAs include carbon-based materials, such as carbon nanotubes (CNTs) [11,12,13,14,15,16,17], graphene [18,19,20,21,22,23], graphite [24,25,26,27,28] and charcoal nanoparticles [29,30,31], and topological insulators, such as molybdenum disulfide (MoS_2_) [32,33,34,35], antimony telluride (Sb_2_Te_3_) [36], Bi_2_Te_3_ [37,38,39], and bismuth selenide (Bi_2_Se_3_) [40,41], etc. In addition to the well-known slow and fast SAs for fiber lasers, recently, germanium (Ge) was also considered as another promising candidate to serve as a fast SA due to its ultrafast recovery time (sub-picosecond degree) and nonlinear saturable absorption [42,43,44]. In previous works, the passive mode-locking laser with the Ge SA was preliminarily explored as early as the 1970s [45,46,47,48]. The saturation intensity and the modulation depth of Ge-based SA were reported as 10 MW/cm^2^ at 10.6 μm and 0.13% at 1.54 μm, respectively [42,45,49]. Although the CO_2_ laser was mode-locked by Ge SA at 10.6 μm in previous works [47], the operational wavelength was far beyond the wavelength required for fiber-optic communications or ultrafast optoelectronic applications in nearby C- and L-band wavelength regimes. Furthermore, the manufacturing process of the saturable Bragg reflector (SBR) structure based on a 40 nm thick Ge film deposited by chemical vapor deposition (CVD) is relatively complex [42]. To date, using the Ge thin-film as a fast SA to initiate the passive mode-locking of the erbium-doped fiber lasers (EDFLs) has not yet been reported until now. In view of the developing progress in saturable absorbers for mode-locked lasers, most of the currently available saturable absorbers include group III-V/II-VI compound semiconductors and other kinds of chemical/topological materials, which can be implemented either through solution-based synthesis with a less-precise control over concentration and thickness, or via high-vacuum molecular beam epitaxy with a higher technical and cost criterion regarding structural design and substrate selectivity. There is no cost-effective approach that can still consider thickness-adjustable and uniformity-controllable issues under substrate-independent synthesis. Plasma-enhanced chemical vapor deposition (PECVD) is an alternative candidate, which fulfills the aforementioned controllability and flexibility for synthesizing saturable absorbers.

In this work, a Ge thin-film SA was synthesized by low-temperature plasma-enhanced chemical vapor deposition (PECVD) upon the SiO_2_/Si substrate and then lifted off from the host wafer. Then, the passively mode-locking EDFL self-started by this Ge thin-film SA was demonstrated. The PECVD-grown Ge thin-film was directly deposited upon the 3 μm thick SiO_2_ layer. The chemical lift-off and transfer process were implemented by a buffered oxide etching (BOE) solution-based etching of the sandwiched SiO_2_ between the Ge and Si wafer. The linear and nonlinear optical transmittances of the Ge thin-film were examined by utilizing a commercially available continuous-wave mode-locked fiber laser at a wavelength of 1570 nm. The material and structural analyses, mode-locking performances, and related parameters of the Ge thin-film were investigated by adjusting the pump power of the EDFL. Lastly, the comparisons of the Ge thin-film and other SAs reported previously were performed.

## 2. Experimental Setup

**Figure 1:**
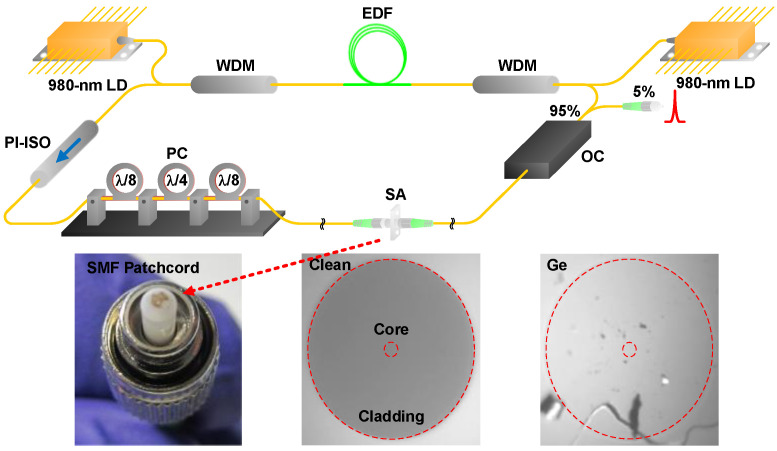
Experimental setup of the passively mode-locked EDFL self-started by the Ge-based SA.

The experimental setup of the Ge thin-film passively mode-locked EDFL is schematically illustrated in Figure 1. The unpolarized fiber laser was constructed with a 2 m long erbium-doped fiber (nLIGHT LIEKKI, Er80-8/125) and a 5.5 m long standard single-mode fiber (SMF, Corning SMF-28). The group velocity dispersion (GVD) of the EDF and SMF were −25.5 and −27.38 ps^2^/km at 1600 nm, respectively, providing the total group delay dispersion (GDD) of −0.202 ps^2^ at 1600 nm for the EDFL. Two wavelength-division-multiplexing (WDM, 980/1585 nm) couplers were utilized to couple the pump power from two 980 nm laser diodes (LDs) to the EDF. A polarization-independent optical isolator was applied to enforce the propagation direction of the EDFL. A polarization controller (PC) was placed into the EDFL cavity with the Ge-based SA to manually adjust the polarization state of the mode-locked pulse. An optical coupler (OC) was installed to output the mode-locked pulse from the 5% coupling ratio port. The Ge-thin-film-based SA was attached to one end-face of the SMF patchcord with an FC/APC connector, which was then sandwiched between the connector end-faces of two SMF FC/APC patchcords. The optical microscope (OM) images showed that the end-face of the fiber was well-covered by the Ge-thin-film-based SA.

**Figure 2:**
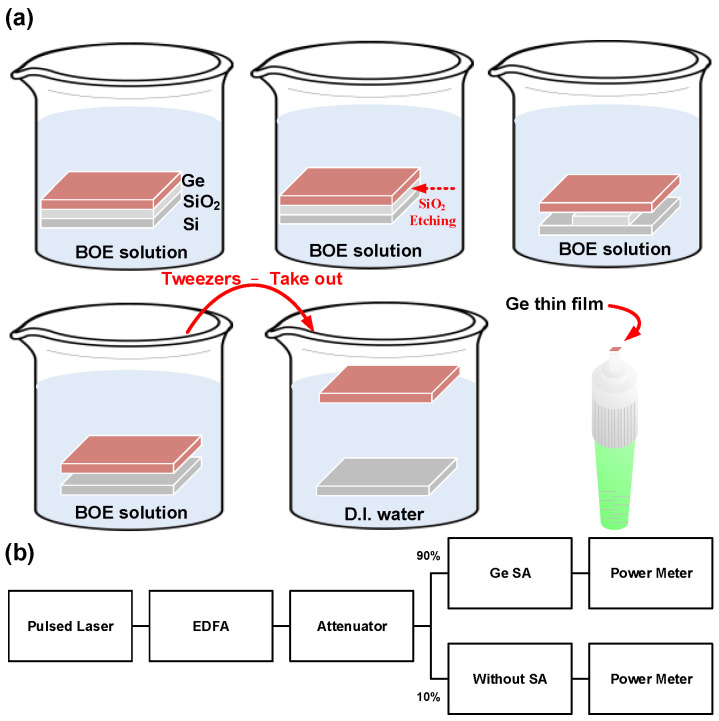
(**a**) The exfoliation process of the Ge thin-film. (**b**) Measured system of the nonlinear transmittance.

For material and structural analyses, the thickness, structural phase, crystallinity, and chemical composition of the PECVD-grown Ge thin-film were analyzed by a field emission scanning electron microscopy (FESEM, JEOL JSM-7001F, Tokyo, Japan), Raman scattering spectroscopy with a continuous-wave argon laser source at 514.5 nm, and a field emission transmission electron microscopy (FETEM, JEOL JEM-2100, Tokyo, Japan). The spectral resolution of the Raman spectrometer was set as 0.75 cm^−1^ in this work. In addition, both the linear and nonlinear transmittances were measured via a commercial mode-locked fiber laser with a central wavelength of 1570 nm, a pulsewidth of 715 fs, and a repetition time of 24.7 ns. The output mode-locked pulse was further amplified by a commercial EDFA and then attenuated by a programmable attenuator by adjusting its output power during intensity-dependent measurements. Regarding the SA sample preparation, the chemical exfoliation process of the Ge thin-film is illustrated in Figure 2a. In the exfoliation process, the Ge thin-film was immersed into the BOE solution to etch out the SiO_2_ layer. The chemical lift-off of the SiO_2_ layer sandwiched between the epitaxial Ge film and Si wafer was implemented in BOE solution. The BOE solution comprised 40% ammonium fluoride (NH_4_F) and 49% hydrofluoric acid (HF). In addition, the NH_4_F/HF ratio was controlled at 6:1. Subsequently, the floated Ge-film was transferred to the fiber patchcord after gradually diluting the BOE solution with the deionized water. Such a specific transfer procedure naturally generated a native oxidized layer with a thickness of less than 10 nm on the Ge surface, which was dipped in the deionized water afterwards to prevent further surface oxidation. Then, the Ge film was transferred from the Si substrate to the deionized (D.I.) water surface. After diluting the residual acid solution, the floating Ge thin-film was attached to the end-face of an FC/APC connector at the end of the SMF patchcord by simply dipping an SMF patchcord into the D.I. water tank with the floated Ge thin-film. Finally, the SMF patchcord with wet Ge thin-film attached to its end-face was baked in an oven at 120 °C for 10 min and naturally dried under room temperature for about 24 h. Then, an optical coupler with a coupling ratio of 10%/90% was used to couple the 90% light into the SMF patchcord with sandwiched the Ge thin-film SA. In contrast, the 10% light was sent into another SMF patchcord without SA for reference, as shown in Figure 2b. Lastly, the performance of the EDFL pulse, mode-locked by the Ge-thin-film-based SA, was analyzed by an optical spectrum analyzer (OSA, Ando, AQ6317B, Kawasaki, Japan) and an intensity autocorrelator (Femtochrome, FR-103XL, Berkeley, CA, USA).

## 3. Results and Discussion

**Figure 3:**
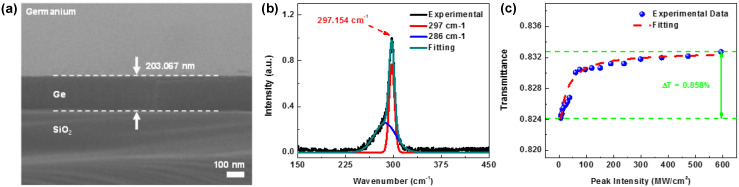
(**a**) FESEM images and (**b**) the Raman spectrum (**c**) the nonlinear transmittance of the germanium thin-film deposited by the PECVD.

The FESEM image of the PECVD-grown germanium thin-film is shown in Figure 3a. The thickness of the Ge layer deposited upon a 3-μm-thick SiO_2_-coated Si substrate was estimated as 203.07 nm. With a near-threshold plasma power during PECVD growth, the deposition time was as long as 15 min for smooth and slow Ge deposition. This deposition rate was as low as 13.5 nm/min at a substrate temperature of 100 °C. For structural analysis, the Raman spectroscopy was implemented to measure the crystallinity of the deposited Ge layer, as shown in Figure 3b. After exciting the surface via an argon continuous-wave (CW) laser at 514.5 nm, the Raman scattering peak of the deposited Ge layer was located at 297.15 cm^−1^ at room temperature [50,51]. This relatively sharp peak with a huge intensity was comparable with the standard crystalline Ge reference centered at 300 cm^−1^. This result indicates the high level of crystallinity for the low-temperature PECVD-grown Ge layer. One Raman scattering peak at 297 cm^−1^ originated from the transverse-optical (TO) mode of the crystalline Ge [52], and nanocrystalline Ge contributed to another Raman scattering peak at 286 cm^−1^ [53]. For the Ge deposited on the SiO_2_/Si substrate, the slight deviation of 3 cm^−1^ was red-shifted from the standard reference at 300 cm^−1^. The deposited Ge film on SiO_2_/Si substrate still suffered from a slight strain [50,51]. Subsequently, the nonlinear optical properties of the Ge film on SiO_2_/Si were lifted off and investigated by utilizing a commercial mode-locked fiber laser with continuously varying illumination power on the sample surface. As a result, the nonlinear transmittance T of the lift-off Ge film is shown in Figure 3c, which can be fitted by using the following equation [24,29,54]:

(1) $$T=\exp\left(-\alpha_{lin}l-\frac{\alpha_{non}l}{1+I_{in}/I_{sat}}\right),$$

where *α_lin_l* and *α_non_l* denote the linear and the nonlinear absorbances, *I_in_* the illumination intensity, and *I_sat_* the saturation intensity of the Ge film. The transmittance of Ge film increases from 0.824 to 0.833 with a ∆T/T of 0.858% when the peak intensity of the mode-locked laser pulse enlarges from 6.2 to 593.5 MW/cm^2^. The linear and the nonlinear absorbance and the peak saturation intensity of the Ge film are *α_lin_l* = 0.183, *α_non_l* = 0.013, and *I_sat_* = 0.8 MW/cm^2^, respectively. Under the input intensity, far below the saturation intensity of the SA, the linear and nonlinear absorption coefficients of *α_lin_* and *α_non_* can be further simplified to *α_lin_* + *α_non_* (1 − *I_in_*/*I_sat_*). In addition, the characteristic *α_non_l*/*I_sat_* can be defined as the self-amplitude modulation (SAM) coefficient (*γ_SAM_*) to fairly evaluate and compare the quality of different SAs. The SAM coefficient is essential for a sufficient modulation depth to initially increase the mode-locked pulse intensity. Therefore, the desirable SA exhibits a low *α_lin_* and high *α_non_* to obtain the low mode-locking threshold and the high modulation depth, especially when considering the SAM coefficient [55,56,57]. In the generalized master equation, the linear absorbance term in the *γ_SAM_* is compensated with the intra-cavity gain such that the mode-locking force is mainly attributed to the nonlinear (saturable) absorbance.

Table 1 shows the nonlinear saturable absorption parameters of the Ge SA and other carbon-based SAs to compare their performance [55]. Compared to other carbon-based SAs, the Ge SA exhibits the largest *α_lin_l*, the smallest *α_non_l*, and lowest *M_D_*. These parameters indicate that the EDFL self-started by the Ge-thin-film-based SA reveals a relatively high mode-locking threshold and weak pulse compression ability. At the early stage of the mode-locking state, mode-locked pulse compression depends strictly on the SAM coefficient. The compression factor can be described by *τ_SAM_* = (2*D_g_*/*γ_SAM_*|A_0_|^2^)^1/2^ with *D_g_*, *γ_SAM_*, and A_0_ denoting the gain dispersion, the SAM coefficient, and the amplitude of the mode-locked pulse, respectively. The SAM coefficient should be sufficiently large for a desirable SA to initiate passive mode-locking. This indicates that a high nonlinear absorbance and a low saturation intensity are mandatory parameters for the superior SA [55]. Although the SAM coefficient of the Ge-thin-film-based SA is not the highest SA among all candidates, it is still better than the charcoal and carbon-black nano-particle-based SAs employed for the easier passive mode-locking of the EDFL.

**Figure 4:**
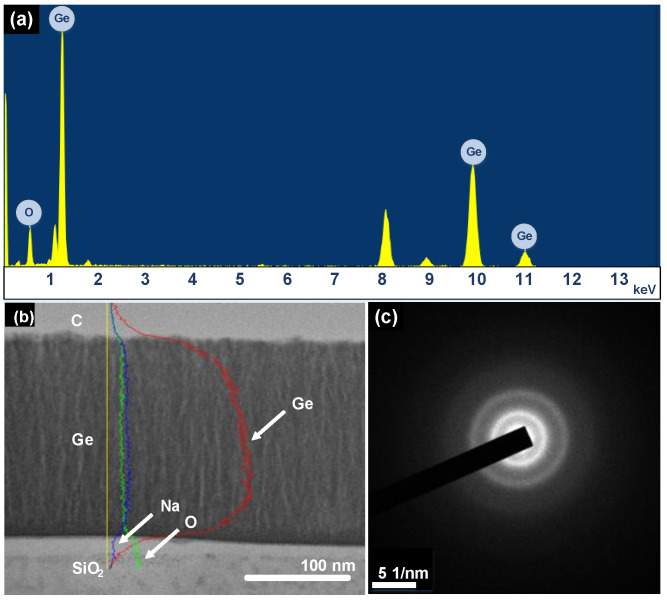
(**a**,**b**) EDS energy and scanning spectra and (**c**) the SAD pattern of the Ge thin-film.

Energy-dispersive X-ray spectroscopy (EDS) and selected area diffraction (SAD) analyses are utilized to characterize chemical composition and crystallinity, respectively, as shown in Figure 4. The EDS analysis confirms the existence of Ge atoms and indicates the presence of residual oxygen (O) atoms, revealing that the Ge thin-film is somewhat oxidized after exposure to the atmosphere for a long time, as shown in Figure 4a. The cross-sectional EDS scanning profile and scanning transmission electron microscopy (STEM) image show three distributional profiles. The background signal related to the sodium (Na) element mainly originates from the SiO_2_ substrate. As observed from Ge- and O-related profiles, the weight percentages of Ge and O are 90% and 10%, respectively, giving rise to the corresponding atomic ratios of Ge and O, which are about 65% and 35%.

**Figure 5:**
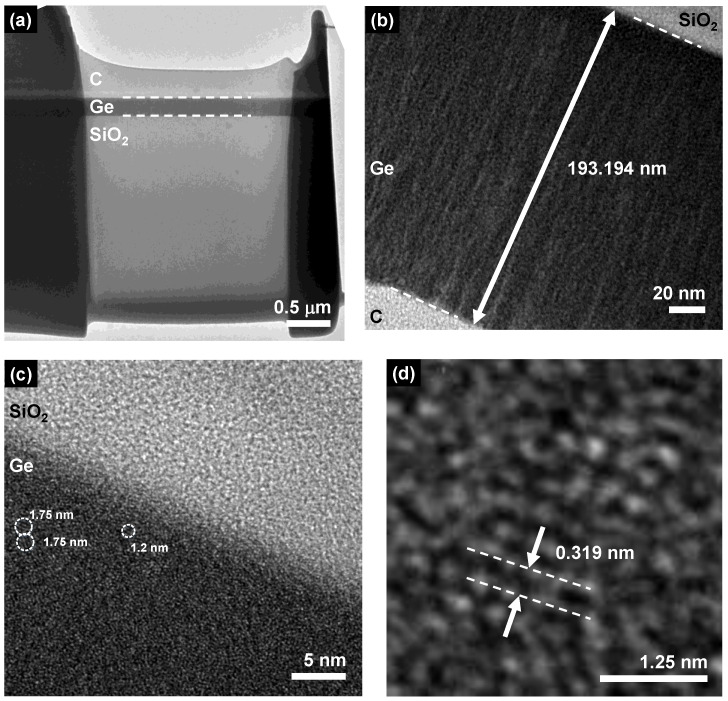
(**a**–**c**) TEM images of the PECVD-grown Ge thin-film at different positions. (**d**) The high-resolution TEM image of the PECVD-grown Ge thin-film.

This EDS result indicates that the Ge thin-film is moderately oxidized because of the residual oxygen contamination during the manufacturing process, as shown in Figure 4b. Furthermore, the SAD pattern reveals two diffraction rings with corresponding radii of 0.317 nm and 0.173 nm. These corresponding radii of diffraction rings are similar to the lattice constants of 0.33 nm and 0.17 nm to indicate the predominated nanocrystalline phases at (111) and (311) orientations in the Ge film [58]. The SAD analysis supports the existence of Ge nanocrystals, as shown in Figure 4c. To observe the crystal structure of the Ge thin-film deposited by PECVD, FETEM is employed with a high-resolution (HR) observation mode, as shown in Figure 5. After slicing the Ge thin-film sample by focused ion beam (FIB) and mounting it on a copper grid, the low-magnification TEM image is shown in Figure 5a. The low-resolution TEM image shows two clear interfaces among the carbon (C) thin-film, Ge thin-film, and SiO_2_ substrate. The thickness of the Ge thin-film is estimated as 193.19 nm, which is in good agreement with the thickness of 203 nm estimated by FESEM, as shown in Figure 5b. In the TEM image, most of the area of the Ge thin-film is nearly an amorphous structure. However, a few Ge nanocrystals exist within the film, as shown in Figure 5c. The size of a few Ge nanocrystals ranges from 1.2 nm to 1.75 nm in the Ge host matrix. In addition, the high-resolution TEM image exhibits the Ge nanocrystal with a layer spacing (d) of 0.319 nm. This shows a good agreement with the reference value of 0.33 nm at (111) orientation, indicating that the growth direction of the Ge nanocrystal is the (111) orientation [54], as shown in Figure 5d.

**Figure 6:**
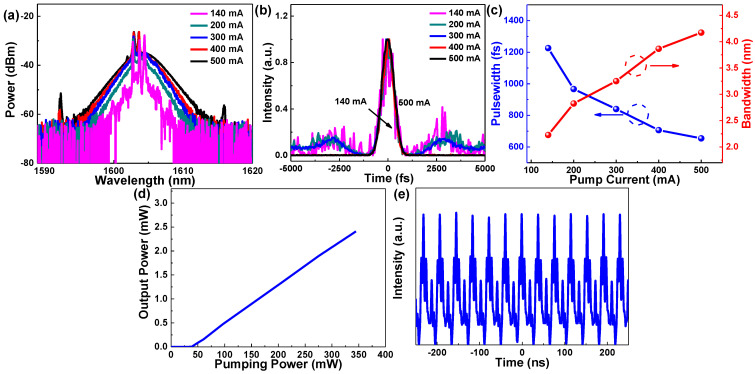
(**a**) Autocorrelated traces and (**b**) corresponding optical spectra of the mode-locked pulse self-started by the Ge-based SA. (**c**) Pulsewidth and bandwidth of the mode-locked pulse self-started by the Ge-based SA under different pumping currents. (**d**) P–I curve and (**e**) pulse train of the mode-locked pulse self-started by the Ge-based SA.

The polarization-dependent optical isolator (PD-ISO) and in-line polarizer (ILP) were not used to construct the passively mode-locked EDFL. Therefore, the mode-locking operation of the EDFL self-started by the NPR effect was excluded as the modeling mechanism [18,59]. Before inserting the Ge thin-film saturable absorber into the EDFL cavity, no self-started mode-locking occurred, even when adjusting polarization by tuning the orientations of three paddles of PC or by increasing the pump current of both 980 nm LDs up to 500 mA. By properly setting the orientations of three paddles of PC, the mode-locking operation, purely self-started by the Ge thin-film-based SA, can be easily achieved by increasing the pump current of the EDFL beyond 140 mA (the mode-locking threshold). The power-dependent evolution of the mode-locked pulse characterized by the autocorrelation traces, the optical spectra, and the trend of soliton pulsewidth and linewidth with an increasing pumping current are shown in Figure 6. The hyperbolic-secant (Sech^2^)-fitted optical spectrum centered at 1604.09 nm enlarges its 3 dB spectral linewidth from 2.23 to 4.17 nm when continually increasing the pump current from 140 to 500 mA, as shown in Figure 6a. The sharpened Kelly spectral sidebands, aside from the mode-locking spectrum, strengthen when the pumping power exceeds 400 mA. This effect reveals that the mode-locked pulse further reshapes into a typical soliton in the negative dispersion regime [60,61]. Some residual CW components are observed in the low-power pumped optical spectra. The Ge-based SA fails to suppress the residual CW lasing component even when carefully adjusting the PC. This phenomenon is dominated by the weak saturable absorption of Ge-based SA. The pulsewidth of the mode-locked pulse is determined with a time-bandwidth product (TBP) of nearly 0.32. This indicates that a slight chirp is associated with the EDFL pulse. Its pulsewidth shortens from 1226 fs to 654 fs when gradually increasing the pump current from 140 to 500 mA, as shown in Figure 6b,c. Both optical spectra and autocorrelated traces fluctuate during the analysis, implying that the Ge thin-film-initiated mode-locking is not stabilized at low pump conditions. The repetition rate is 27.3 MHz and the maximal output power is 2.4 mW under a set of 5% coupling from the EDFL cavity, as shown in Figure 6d,e. Regarding the limitations of operating the SA at the current stage, the pumping power required at the mode-locking threshold is 97 mW. The upper pumping limitation of the EDFL is set by the optical damage threshold of the SA at about 0.2 J/cm^2^ [62], which is far beyond the operating condition with an intra-cavity optical pulsed energy intensity of <2 mJ/cm^2^ under the current pumping case.

Lastly, Table 2 summarizes the related parameters to further evaluate and compare the passive mode-locking performances of the EDFL self-started by versatile group IV; semiconductor-based SAs (including the Ge thin-film and the carbon-based materials mentioned above) [55]. With the central wavelength of the mode-locked EDFL located between 1570 and 1600 nm, the narrowest pulsewidth and the longest 3 dB spectral linewidth of the nearly transform-limited soliton pulse from the EDFL, passively mode-locked by Ge thin-film SA, are 654.39 fs and 4.17 nm. In comparison, the carbon-based SAs show better results for their pulsewidth and linewidth values, ranging from 305 to 435 fs and from 8.05 to 6.04 nm, respectively. The EDFL pulses mode-locked by charcoal, carbon-black, and Ge SAs reveal larger TBP than graphene, graphite, and graphene-oxide SAs. This indicates that the soliton obtained by former SAs still exhibits a chirped pulse. Even though Ge thin-film SAs suffer from severe oxidation that weakens its mode-locking force, the SAM in the EDFL can still be started to facilitate the initiation of self-phase modulation (SPM) at the second stage of compressing the soliton into the hundreds-of-femtoseconds regime.

Table 3 shows the nonlinear absorption and mode-locking parameters for the Ge thin-film and Ge nano-sheet SAs [63]. Although the modulation depth and nonlinear absorbance of the Ge thin-film in this work are slightly smaller than those of the Ge nano-sheet, the Ge thin-film still provides a larger SAM coefficient and smaller satu-rated intensity, demonstrating a shorter pulsewidth and wider 3 dB bandwidth. In ad-dition, the easy fabrication of the Ge thin-film also facilitates a larger tolerance of sub-strate selection. These performances confirm the Ge thin-film as one of the next-generation SA candidates for passively mode-locked fiber lasers.

## 4. Conclusions

The passive mode-locking of the L-band EDFL at 1600 nm initiated by low-temperature PECVD-grown Ge-thin-film-based SA is demonstrated. The Ge thin-film is directly synthesized by PECVD upon the SiO_2_-covered Si substrate at temperatures as low as 100 °C. In addition, this film can be chemically exfoliated by a BOE solution and then transferred onto the end-face of an FC/APC connector in the SMF patchcord. The 200 nm thick Ge-thin-film-based SA was formed by sandwiching it with two identical FC/APC connectors and inserting it into the unpolarized EDFL cavity. A structural analysis with Raman scattering spectroscopy reveals a sharp peak at 297 cm^−1^ and a left-side pedestal at 286 cm^−1^. This indicates that the Ge thin-film exhibits a high poly-crystallinity with buried nano-clusters. The SAD pattern shows two diffraction-ring radii of 0.317 and 0.173 nm related to the dominated phase at (111) and (311) orientations. The zoom-in HRTEM image confirms that the Ge thin-film exhibits some nanocrystals with a d-spacing of 0.319 nm corresponding to the crystal phase at (111) direction, as approved by the SAD results. The linear and nonlinear transmittances of the Ge thin-film are characterized as T = 0.82 and T = 0.86, leading to the characteristic SAM coefficient of 0.016 for the Ge thin-film. Enlarging the pump current from 140 (the mode-locking threshold) to 500 mA obtains a nearly transform-limited soliton pulsewidth, shortening from 1.23 ps to 654 fs. The 3 dB spectral linewidth is concurrently broadened from 2.23 to 4.17 nm to give a slightly chirped TBP of 0.32. Compared with the carbon-based SAs, the Ge-thin-film-based SA provides a weaker mode-locking force and suffers from oxidation rather than graphene. However, it already behaves better than nano-scale charcoal or carbon-black particles in initiating the SAM-based mode-locking in the EDFL at the first stage.

## Figures and Tables

**Table 1:** Comparison of the nonlinear absorption parameters for the Ge SA and previous works [55].

Materials	*α_lin_l*	*α_non_l*	*I_sat_* (MW/cm^2^)	*M_D_* (%)	*γ_SAM_*
Few-layer graphene	0.033	0.054	0.8	60	0.068
Graphite nano-particle	0.047	0.057	1.15	55	0.05
Graphene oxide nano-particle	0.068	0.059	1.55	45	0.038
Carbon black nano-particle	0.092	0.048	3.52	30	0.014
Charcoal nano-particle	0.099	0.037	6.1	23	0.006
Ge thin-film	0.183	0.013	0.805	5.358	0.016

**Table 2:** Comparison of the mode-locking performances of the Ge SA and our previous work [55].

Materials	*λ_peak_* (nm)	Pulsewidth (fs)	3-dB Spectral Bandwidth (nm)	TBP
Few-layer graphene	1572	305	8.05	0.315
Graphite nano-particle	1571	335	7.51	0.315
Graphene oxide nano-particle	1571	370	7.05	0.315
Carbon black nano-particle	1570	415	6.49	0.32
Charcoal nano-particle	1570	435	6.04	0.32
Ge thin-film	~1600	654	4.17	0.32

**Table 3:** Comparison of the nonlinear absorption and mode-locking parameters for the Ge SA and Ge nano-sheet [63].

Materials	*α_lin_l*	*α_non_l*	*I_sat_* (MW/cm^2^)	*M_D_* (%)	*γ_SAM_*	*λ_peak_* (nm)	Pulsewidth (fs)	3-dB Bandwidth (nm)	TBP
Ge nano-sheets	≈0.09	0.119	2266	13.9	5.3 × 10^−5^	1550	901	3.2	0.34
Ge thin-film	0.183	0.013	0.805	5.36	0.016	1600	654	4.17	0.32

## Data Availability

The data presented in this study are available on request from the corresponding author.
